# COVID- 19 and sickle cell disease: autopsy findings of three deaths at the 37 Military Hospital, Accra, Ghana

**DOI:** 10.11604/pamj.2022.41.332.30035

**Published:** 2022-04-25

**Authors:** Seth Andrews Attoh, Emmanuel Sarkodie, Raymond Fatchu, Amma Benneh-Akwasi Kuma, Edward Asumanu, Mary McAddy, Alfred Toppar, Frederick Hobenu, Kwasi Agyeman-Bediako, Lawrence Edusei, Patrick Kafui Akakpo

**Affiliations:** 1 Department of Anatomical Pathology, 37 Military Hospital, Accra, Ghana,; 2 Department of Haematology, 37 Military Hospital, Accra, Ghana,; 3 Department of Clinical Pathology, 37 Military Hospital, Accra, Ghana,; 4 Department of Haematology, University of Ghana Medical School, Korle-Bu Teaching Hospital, Accra, Ghana,; 5 Postgraduate Unit, 37 Military Hospital, Accra, Ghana,; 6 Medical Division, 37 Military Hospital, Accra, Ghana,; 7 Department of Pathology, Korle-Bu Teaching Hospital, Accra, Ghana,; 8 Department of Pathology, School of Medical Sciences, University of Cape Coast, Cape Coast Teaching Hospital, Cape Coast, Ghana

**Keywords:** COVID-19, autopsy findings, Ghana, sickle cell disease, SARS-CoV-2, pathology

## Abstract

The main pathological effects of COVID-19 infection have been reported to occur in the lungs, with the most pronounced manifestation being reported as Adult Respiratory Distress Syndrome (ARDS) with thromboembolic phenomena. Sickle Cell Disease (SCD) is a common genetic disorder present in 2% of newborns in Ghana. The complications of SCD include Vaso-Occlusive Crisis and Acute Chest Syndrome, which primarily manifest in the lungs. The effects of SCD on the progression of COVID-19 have not been extensively and clearly documented in literature. The objective was to describe the clinical and pathological findings in three SCD patients who died of COVID-19 related complications. A complete autopsy was performed on each of the three SCD patients who were presumed to have COVID-19. Lung swabs were subsequently taken and tested for SARS-CoV-2. The differences in histopathological findings of the three cases were highlighted and correlation with clinical findings was also done. Lung histopathological findings for all three cases were consistent with Acute Respiratory Distress Syndrome (ARDS)/ Diffuse Alveolar Damage (DAD) described for infections with COVID-19 and lung swabs tested for SARS-CoV-2 by real time Reverse Transcription Polymerase Chain Reaction (rRT-PCR) were positive. Though SCD has been reported not to adversely affect an individual´s chance of worse outcome when infected with COVID-19, our findings suggest otherwise. We suggest that SCD may be an important co-morbidity that needs to be considered in COVID-19 patients and when present needs to be considered as an adverse risk for poor outcomes. Also, post-discharge anti-coagulation and monitoring should be encouraged. More autopsies are required to fully understand the pathogenesis of COVID-19 in SCD patients.

## Introduction

Novel Coronavirus Disease 2019 (COVID-19) is caused by Severe Acute Respiratory Syndrome-Corona Virus-2 (SARS-CoV-2) an RNA virus which belongs to the Coronavirus family [1]. The main pathological effects of COVID-19 infection have been reported to occur in the lungs, with the most pronounced/the severest manifestation reported as Adult Respiratory Distress Syndrome/Diffuse Alveolar Damage (ARDS/DAD) [2, 3]. In addition, many patients present with thromboembolic phenomena. Sickle Cell Disease (SCD), affecting about 2% of newborns in Ghana, is the generic term for the group of haemoglobinopathies caused by the occurrence of Haemoglobin S (HbS) in the Homozygous Form-Sickle Cell Anaemia (HbSS) or as the heterozygous combination of HbS with another abnormal haemoglobin such as Haemoglobin C (HbC) or beta-thalasaemia (HbSb-thal) [4]. SCD is the most common hereditary haematological disorder and is associated with increased morbidity and mortality. It is a condition characterized by sickling of red blood cells in circulation with resultant occlusion of vessels that may result in complications in various organ systems of the body [4, 5]. It has been reported that COVID-19 infection can exacerbate pulmonary complications in SCD patients, especially in those who already have pulmonary complications such as Acute Chest Syndrome, Pulmonary Hypertension and ARDS. This results in an increase in morbidity and mortality [3]. Acute Chest Syndrome (ACS), a term used to cover conditions characterized by chest pain, cough, fever, hypoxia (low oxygen level) and new lung infiltrates may be the result of sickling in the small blood vessels in the lungs causing pulmonary micro-thromboembolism and subsequent infarction with added viral or bacterial pneumonia [5, 6]. SCD patients may have respiratory complications as a result of vaso-occlusive crisis. Complications include ACS with associated Pulmonary Hypertension [5, 6].

A handful of case reports of SCD patients with vaso-occlusion and/or ACS caused by SARS-CoV-2 suggest that awareness of the relationship between COVID-19 infection and SCD and the provision of appropriate intervention when both are present, can result in excellent recovery [5, 6]. This may not necessarily be true, as we observed 3 cases of SCD mortalities with COVID-19 within 4 months. It has been suggested that there are important associations between ACS and COVID-19 however literature that describes the effects of SARS-CoV-2 infection in patients with SCD are limited. SARS-CoV-2, which is a respiratory virus, causes pneumonia and this can lead to episodes of ACS and potentially respiratory failure in a patient with SCD resulting in increased mortality. ACS is a leading cause of intensive care admissions and mortality in SCD patients [7]. Postmortem examination is an invaluable tool in understanding the pathobiology of disease. The causes of death from COVID-19 range from acute respiratory distress syndrome (113; 100%), type I respiratory failure (18/35; 51%), sepsis (113; 100%), acute cardiac injury (72/94; 77%), heart failure (41/83; 49%), alkalosis (14/35; 40%), hyperkalemia (42; 37%), acute kidney injury (28; 25%), and hypoxic encephalopathy (23; 20%) [8]. The majority of COVID-19 patients have macroscopic and/or microscopic thrombi at autopsy [2]. COVID-19 has a 58% incidence of deep venous thrombosis, of which 33% of patients are reported to have had pulmonary embolism as a direct cause of death [9]. This case series therefore describes the clinical and pathological findings in three SCD patients who died of COVID-19 related complications.

## Methods

Using similar methodologies adopted by Attoh *et al*. [2], a complete autopsy was performed for each of the three SCD patients who were presumed to have COVID-19 between September 2020 and January 2021. Lung swabs were subsequently taken and tested for SARS-CoV-2. The differences in histopathological findings of the three cases were highlighted and correlation with clinical findings was also done.

## Results

### Case 1

A 59-year-old male with SCD (HbSS genotype) presented with a 5-day history of general body weakness, loss of appetite, a day´s history of lethargy and confusion. Random blood sugar (RBS) at presentation was 33.3mmol/L. Three weeks prior to presentation, he was admitted for five days with complaints of fever, chills, loss of appetite and general weakness. He had denied cough, bone pain, anosmia or sore throat at that time. His Fasting Blood Sugar (FBS) was normal. A Computed Tomography (CT) scan of the chest revealed bilateral peripheral ground-glass opacities of apical and basal regions of the lungs suggestive of COVID-19, but real-Time Reverse-Transcription Polymerase Chain Reaction (rRT-PCR) was negative for SARS-COV-2 on naso-pharyngeal swab sample. He was however treated for COVID-19 (dexamethasone, ceftriazone, azithromycin, hydroxychloroquine and dalteparin) and discharged to self-isolate at home. On examination at this presentation, the patient was observed to be confused, not pale, anicteric, dehydrated, febrile (temperature- 38.3 degrees celcius) and was observed to have a herpes labialis. He had a respiratory rate of 22 cycles per minute and oxygen saturation of 94% on room air. The air entry was adequate bilaterally and breath sounds were vesicular with no added sounds. There were no abnormalities detected in the other systems. The patient was managed for Diabetes Mellitus (DM) to rule out sepsis. Other laboratory investigations revealed Haemoglobin (Hb) of 6.3g/dl, White Blood Cell (WBC) count of 17.1 X 10^9^/L and of Platelets 68 X 10^9^/L. He was managed on Insulin, Ceftriazone and Dalteparin. The patient improved on the second day of admission but in the evening of the third day he started de-saturating with Saturated Pressure of Oxygen (SPO2) of 89% on room air. Supplemental oxygen via nasal prongs was initiated. On the fourth day whiles still on the oxygen (SPO2 of 96%) he complained of severe back pain hence an impression of Vaso-Occlusive Crises (VOC) was made and he was managed accordingly. The back pain improved significantly by the morning of the next day, however later that day the patient developed shortness of breath and went into respiratory distress. An impression of ACS precipitated by VOC to rule out pulmonary thromboembolism was made requiring ventilation. A repeat nasopharyngeal swab for SARS-CoV-2 testing was to be collected that day but the patient passed before the sample could be taken.

**Figure 1:**
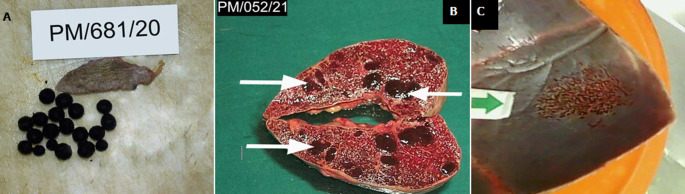
gross photographs of SCD patients with COVID-19 at autopsy (37 Military Hospital, Accra, Ghana, September 2020 to January 2021); A) autosplenectomy with pigment gall stones; B) regenerative nodules of extramedullary haematopoises in spleen (white arrows); C) thinning of cortical bone of skull with bone resorption (green arrow)

**Figure 2:**
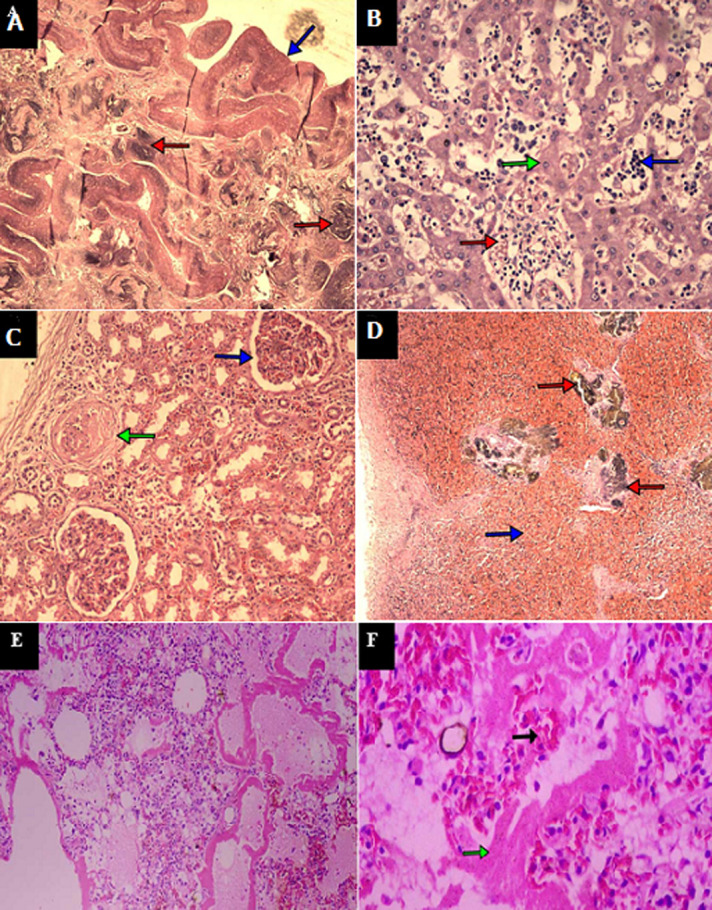
haematoxylin & Eosin (H&E) staining of various tissues of SCD patients with COVID-19 (37 Military Hospital, Accra, Ghana, September 2020 to January 2021); A) advanced autosplenectomy with calcified siderofibrotic nodules (red arrows) X 100; B) liver sinusoids (green arrow) with erythrophagocytosis of RBCs (red arrow) and extramedullary haemopoiesis (blue arrow) X 100; C) normal (blue arrow) and fibrosed glomerulus (green arrow) X 100; D) sidero-fibrotic nodules (red arrow) interspersed with vessels containing sickled RBCs (blue arrow) X 100

Autopsy findings showed an adult male with a puffy face who was pale, not jaundiced, with no bipedal edema. There was no peripheral lymphadenopathy. There were stigmata of SCD i.e., bossing of the frontal and parietal bones of the skull, loose arrangement of the maxillary teeth and long upper and lower limbs (extremities). There was bilateral serous plural effusion (R 200mls, L 220mls). The larynx, trachea and bronchi showed frothy secretions. The lungs, right weighing 795g and the left weighing 700g were heavy, firm, congested with areas of patchy consolidation. There were pulmonary thromboemboli within the medium-sized pulmonary arteries. The aorta showed atheromatous plaques, with the heart which weighed 490g showing concentric Left Ventricular Hypertrophy in addition to a Right Ventricular Hypertrophy. The chambers, valves and other musculature appeared grossly normal. The coronary arteries showed moderate atherosclerosis. The spleen was barely visible (auto splenectomy) measuring 5x3x1cm and weighed 7g (Figure 1A). There was no intra-abdominal lymph node enlargement. The gastrointestinal system showed moderate ascites (300mls). Significant findings were an enlarged liver weighing 1740g with appearances of early micro nodularity and multiple pigment stones in the gall bladder (Figure 1A). All the other organ systems appeared grossly normal. Histopathological examination of the lungs showed interstitial fibrosis with mixed inflammatory cell exudates in the alveolar spaces including multinucleated giant cells. There was Diffuse Alveolar Damage (DAD) with prominent Hyaline Membrane (HM) formation. There was however no sickling of the red cells observed in the lungs. There were however microthrombi within the smaller pulmonary vessels at the periphery of the lungs. The splenic architecture was completely destroyed with dense masses of yellowish-brown pigment enmeshed in fibrous tissue with areas of calcification. Areas of sidero-fibrotic nodules interspersed with vessels containing sickled erythrocytes were noted (auto-splenectomy) (Figure 2A). The liver showed sinusoidal dilatation and congestion with sickled erythrocytes as well as erythrophagocytosis by Kupffer cells (Figure 2B). Scattered fibrosed glomeruli as well as thrombosis of arterioles were noted in the kidney (Figure 2C). A postmortem lung swab sample taken to repeat the rRT-PCR for SARS-COV-2 was positive. The cause of death was stated as acute pulmonary thromboembolism/ACS due to or as a consequence of COVID-19 infection with secondary bronchopneumonia in a Sickle Cell Disease (HbSS genotype) patient. A summary of clinical and pathological findings is presented in Table 1.

**Table 1:** summary of clinical and pathological findings of 3 SCD patients at the 37 Military Hospital, Accra, Ghana

ID Sex Age (yrs)	Presentation	Clinical Signs	Co-morbidities	Results of imaging	Results of Lab investigations	Clinical diagnosis and treatment	Significant autopsy findings and Treatment	Cause of death
1. M 59	Generalised weakness, confusion, and lethargy	Confused, lethargic, febrile,respiratory distress	SCD (SS) DM	Bilateral ground-glass opacities of apical and basal regions	Elevated blood glucose (33.3mmol/L) Hb 6.3g/dl, WBC 17.1 X 109/L, Platelets 68 X 109/L Antemortem COVID-19 test negative	SCD (SS) with VOC and DM Azithromycin, Dexamethazone, Ceftriazone, Dalteparin, Hydroxychloroquine,Insulin Acyclovir	Severe congestion and oedema of lungs with patchy consolidation, DAD with HM formation, microthrombi in lungs, stigmata of SCD externally, autosplenectomy, Hepatomegaly, choleilithiasis, Bi-ventricular hypertrophy	COVID-19 pneumonia with secondary bacterial infection in Sickle cell anaemia
2.M 12	Fever, difficulty in breathing, sore throat, chest pain	Fever, reduced oxygen saturation	SCD (SS)	Ground-glass appearance on chest X-ray	Hb 4.8g/dl WBC 12.62 x109L RBS 24.5 mmol/L and COVID-19 Negative	SCD (SS) with DKA Insulin, Ceftriazone, Ciprofloxacin, Azithromycin, Clindamycin, Arthemeter-lumifantrine	Firm and oedematous lungs, DAD with HM formation, microthrombi in pulmonary vessels, Stigmata of SCD externally, fibrosed spleen with extramedullary haematopoiesis, Hepatomegaly, cholelithiasis, Bi-ventricular hypertrophy, thinning of bones of the skull	COVID-19 pneumonia with secondary bacterial infection in Sickle cell anaemia
3. F 32	Difficulty in breathing	Respiratory distress, bronchial breadth sounds with basal crepitations	SCD(SC), C/S on account of uterine fibroids, gestational DM, anaemia	Ground-glass opacification on CT scan	Hb 4.7g/dL, WBC 38.56x109L RBS SCD (SC) Negative antemortem COVID-19 test	Bilateral pneumonia, COVID-19. Started on Ceftriaxone, Azithromycin, Dexamethasone,Dalteparin	Firm and oedematous lungs, DAD with HM formation, microthrombi in pulmonary vessels, Type 2 pneumocyte hyperplasia, Hepatoslenomegaly, Erythrophagocytosis in liver and spleen, renal papillary necrosis	COVID-19 pneumonia in a patient with SCD, status post cesarian section for uterine fibroids

### Case 2

A 12-year-old boy with sickle cell anaemia (HbSS genotype) presented with difficulty in breathing and general body pains. He presented a month earlier to a peripheral facility and was referred as a case of SCD (SS) with acute chest syndrome, but delayed in reporting due to financial difficulties. At presentation, his temperature was 38°C. He was pale but not jaundiced and weighed 24.3kg. His RBS was 6mmol/L and had a heart rate of 120bpm with a grade 3 systolic murmur at the apex. Respiratory rate was 32 cycles per min, air entry was reduced, breath sounds were vesicular with no added sounds. The liver was enlarged and tender and was at 5cm below the costal margin. He had an ulcer measuring 2.0x2.0cm at the left medial malleolus. Laboratory investigations revealed an initial Hb of 5.4g/dl with a WBC of 12.62x109/L, later on day 10 the Hb was 4.8g/dl the WBC was 21.18x109/L. He was started on Intravenous (IV) Ceftriazone 2g daily, IV Clindamycin 120mg and haematinics. He was also transfused with 360mls of packed red cells on days 2, 4, 9 and 10 of admission and adequately hydrated on oral and IV fluids. He was given IV Furosemide 25mg daily after transfusion. He however became breathless on day 6 with flaring of the ala nasae. Air entry was reduced. SPO2 was 93% in room air and 98% on 2L/minute oxygen therapy via intranasal prongs. He had a persistent fever and chest X-ray done showed bilateral patchy peripheral ground - glass opacities suggestive of COVID-19 pneumonia. Tab Azithromycin 250mg daily, IV ciprofloxacin 250mg twice daily were added to his medications while the Ceftriazone discontinued. He was also given Artemeter/Lumefantrine and hydrocortisone 100mg four times daily. Blood culture, HIV screen and COVID - 19 tests were ordered, but all came out negative. On day 8, he looked sick, dehydrated, weak and refused meals. He was tachypnoeic (respiratory rate 32 cycles per min) with reduced air entry and bronchial breath sounds. He became irritable and later fell unconscious while SPO2 dropped to 88% on 7L/min of oxygen. Urine dipstick done on day 10 showed leucocyte 2+, protein 2+, glucose 2+ and ketones 4+. RBS was now 19.1 mmol/L but rose to 25.4 mmol/L. After a diagnosis of diabetes with Ketoacidosis was made, he was started on insulin, but he had a cardiac arrest on day 11 at the Intensive Care Unit (ICU) and died after failed resuscitation.

Autopsy showed the body of a male child with stigmata of SCD. He was pale and cyanosed but not jaundiced and had no bipedal edema. There was an ulcer measuring 2cm across on the left medial malleolus. The larynx, trachea and bronchi showed frothy secretions. The lungs, right weighing 400g, left weighing 350g, were firm and mildly edematous and the pulmonary arteries show mild fatty streaks. The aorta appeared grossly normal, while the heart weighed 300g and showed biventricular hypertrophy. The spleen weighed 19g, was small, measuring 7x3.5x1.5cm, appeared fibrotic and few dark brown regenerative nodules were noted on cut surface (Figure 1B). The esophagus appeared grossly normal, and the stomach contained coffee-ground secretions, while the intestines and pancreas appear grossly normal. The gall bladder showed few pigment stones and the liver weighed 1,950g, was enlarged and congested. There was severe palor of the renal papillae. There was thinning of the cortical bones of the skull with bone resorption (Figure 1C). The other organ systems did not show any significant macroscopic abnormality.

**Figure 3:**
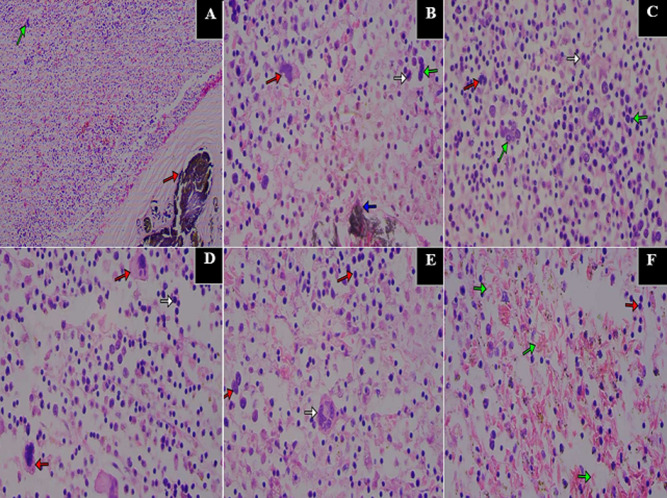
haematoxylin and Eosin (H&E) staining showing extramedullary haemopoiesis in the spleen (37 Military Hospital, Accra, Ghana, September 2020 to January 2021); A) Junction between sidero-fibrotic nodule (red arrow) and haemopoietic cells (green arrow) X100; B) myeloid precursor (red arrow), erythroid precursors (green arrow) and sidero-fibrotic area (blue arrow) X400; C) erythroblasts at various stages of differentiation (green arrows), neutrophil band cell (red arrow) and a mature neutrophil (white arrow) X400; D) myeloid precursors (red arrows) and erythroid precursors (white arrow) X400; E) erythroid precursors (red arrows) and a megakaryocyte (white arrow) X400; F) sickled red blood cells (green arrows) and erythroid precursors (red arrow) X400

Histopathology of the lungs showed DAD with extensive HM bilaterally (Figure 2E and Figure 2F). Macrophages, including giant cells, lymphocytes and plasma cells were seen in the alveolar spaces. There was thickening of the interstitium with fibrosis in areas. Microthrombi were noted at the periphery of the lungs. The spleen showed sidero-fibrotic nodules interspersed with vessels containing sickled RBCs (Figure 2D). Areas of extramedullary haemopoiesis were noted in the regenerative nodules of the spleen on histology revealing erythroid and myeloid precursors, megakaryocytes in a pool of sickled RBCs (Figure 3). The liver showed congested sinusoids with sickled RBCs. Areas of extramedullary haemopoiesis were also noted. The rRT-PCR for SARS- CoV-2 on lung swabs was positive. The cause of death was stated as Diabetes Ketoacidosis (DKA) due to or as a consequence of severe COVID-19 pneumonia in a known SCD (SS) patient. A summary of clinical and pathological findings is presented in Table 1.

### Case 3

A 32 years old female with SCD (HbSC genotype) presented with difficulty in breathing two days after elective Caesarean Section (C/S) on account of uterine fibroids, gestational DM and anaemia. She was noticed to be desaturating on room air in a peripheral facility after onset of symptoms and was referred to the Military Hospital for further management. On examination, she was pale with a tinge of jaundice. She also had grunting respiration with a respiratory rate of 28 cycles per minute. Air entry was reduced in both lungs, with bronchial sounds. Her Hb was 4.7g/dl at presentation, but rose to 6.3g/dl after receiving two pints of packed red blood cells. Other investigations showed a blood pressure of 148/91mmHg, SPO2 of 94% on O_2_ non-rebreather mask, Random Blood Sugar (RBS) was 7.7mmol/L. There was an elevated WBC count of 38.56x10^9^/L with the following differentials; neutrophils (45.2%), lymphocytes (52.3%), basophils (0.7%) and monocytes (1.8%). A CT scan of the chest showed bilateral ground glass opacities, but antemortem nasopharyngeal swab test for SARS-CoV-2 turned out negative. She was however managed with IV Dexamethiazone 8mg, 8 hourly, IV Ceftriazone 2g daily, Tab Doxycycline 100mg bd, SC Dalteparin 15,000 units daily and analgesia. She however died 2 days on admission after worsening breathlessness and desaturation on supplemental oxygen.

Autopsy showed an obese female adult wearing stockings on both legs, pale with a tinge of jaundice and cyanosed centrally. The abdomen was distended with presence of linea nigra while the breasts were engorged with milk expression from the nipples. There was a Pfannenstiel incision with suture in-situ on the lower abdomen. No stigmata of SCD were noticed externally. The larynx, trachea and bronchi show frothy secretions. The lungs (R 820g, L760g) were heavy, firm and congested. There was no pulmonary embolism, but the pulmonary arteries show the presence of very thick dark, shiny clotted blood as well as atheromatous plaques. The aorta showed few fatty streaks. The heart was enlarged, weighing 380g and showed bi-ventricular hypertrophy but no dilatation. The valves and chambers were however grossly normal. The spleen was enlarged, measuring 20x9x7cm. It weighed 870g and appeared heavily congested on cut surface, with indistinguishable Malpighian corpuscles. The kidneys (right weighing 165g, left weighing 170g) appeared edematous and congested with smooth subcapsular surfaces. There was clear cortico - medullary differentiation, but the renal papillae appeared very pale. There was no distortion of the calyces and pelves, but the ureters were dilated. The bladder showed few mucosal hemorrhages. The uterus measured 170mm in length and showed few intramural fibroid nodules, the largest measuring 45mm across. A lower uterine segment suture was present and intact. The myometrium appeared thickened and the endometrial cavity contained blood clots (about 200mls). The liver was enlarged, weighed 1900g and was fatty. There were no stones in the gall bladder.

Histopathology of the lungs showed DAD with HM formation. There were microthrombi in the smaller pulmonary arteries and type 2 pneumocyte hyperplasia was evident. There were areas of interstitial fibrosis, and the alveoli were filled with macrophages, plasma cells and few sickled RBCs. The pulmonary capillaries were congested with sickled red blood cells. The splenic pulp was stuffed with sickled RBCs. There were areas of haemorrhage around compressed Malpighian corpuscles, and erythrophagocytosis was noted as well. The sinusoids of the liver were filled with sickled RBCs and erythrophagocytosis by Kupffer cells were noted. Renal papillary necrosis was evident. Few placental remnants were noted in the endometrium and within thickened myometrial fibers. Postmortem lung swab samples taken to repeat the PCR for SARS-COV 2 were positive. The cause of death was stated as severe COVID-19 pneumonia in a known SCD (HbSC genotype) patient. A summary of clinical and pathological findings is presented in Table 1.

## Discussion

Routine COVID-19 testing in Ghana is conducted using nasopharyngeal and throat swab samples. Results from such manner of sampling have been reported to have higher false negative test rates and hinge strongly on the skill of the samplers [2, 10]. In all three patients, COVID-19 tests based on nasopharyngeal samples taken ante-mortem were falsely negative. The reason being that lung swabs taken during postmortem examination tested positive, and this was further confirmed by pathological findings in the lung of all three cases. The extent to which these false-negative results may have caused delays in the management of these patients as COVI9-19 cases and its contribution to their death cannot be ascertained. In this regard, some proposals for clinical (including radiological) criteria for the diagnosis and treatment of COVID-19 are in order. This may help improve outcome since treatment can commence earlier even in patients with false negative tests [10].

All three cases under review had pathological evidence of lung injury (DAD) which is in line with previous reports [2, 11]. COVID-19 has also been reported to result in thromboembolism. This has been a significant finding in most autopsy-based studies of COVID-19, and it significantly involves the lungs [2, 11]. It has been suggested that identifying and actively managing this complication of COVID-19 in addition to ARDS/DAD will likely improve outcomes [3, 5, 12]. Thromboembolic events are leading causes of deaths in sickle cell disease patients [7, 13]. SCD and COVID-19 patients are both prothrombotic states, thus some experts currently recommend treatment of asymptomatic SCD patients with mild COVID-19 with prophylactic doses of low molecular weight heparin while symptomatic SCD patients with severe disease should be treated with therapeutic doses [14, 15]. The eldest of our three patients (Case 1), aged 59 years, was not on prophylactic anticoagulants after his previous discharge. Post discharge anticoagulation and monitoring of platelet count, prothrombin time, D-dimer and fibrinogen levels could have prevented him from forming a thrombus with subsequent development of a pulmonary thromboembolism. The other lung findings of congestion, heavy and edematous lungs are consistent with other manifestations of COVID-19 disease and also sickle cell related acute chest syndrome [2, 6, 7]. Histologically, no sickling of red cells was observed in the lungs (Case 1) which should have been the case if he had VOC leading to ACS. In this patient, the VOC leading to ACS could probably be due to the thromboembolism. The predisposition to this thromboemboli was as a result of his severe COVID-19 infection and other factors such as prolonged bed rest. The auto splenectomy found at autopsy in case 1 and 2 is in keeping with sickle cell anaemia due to the progressive atrophy of the spleen that results from repeated attacks of vaso-occlusion with infarction [13, 16-19].

Sudden, persistent elevated blood sugar has consistently been reported in individuals who die from COVID-19. Diabetes has also been consistently reported as an important co-morbidity associated with worse outcome in COVID-19 infections [2, 12]. Two of the patients (case 1 and 2), who were not known diabetics, were managed for persistently elevated RBS. Though case 3 had gestational diabetes and her RBS was within normal limits, hyperglycemia is a finding in many reports on severe COVID-19 infection. It appears that RBS elevation is associated with severe COVID-19 and may worsen the outcome of the disease. It is likely to have had a negative impact on patients with COVID-19 infection, as has been seen in earlier case series [2, 12, 13]. Further, studies are recommended to understand whether COVID-19 is a factor in the development of DM. Presenting features of COVID-19 and SCD complications can overlap significantly, and a high level of vigilance is needed while providing care to patients with SCD especially during the pandemic period [3, 5]. It is conceivable that if both pathologies coexist in a patient, an enhanced inflammatory cascade is expected.

However, such changes have not been reported and have not resulted in less favorable outcomes among SCD patients with COVID-19. Anecdotally, the proportion of SCD patients in Ghana and the number of case reports on deaths of SCD patients due to COVID-19 infection suggest that they may not be at increased risk despite the profound effects of both pathologies on the lung [5]. A study of the progression of COVID-19 in a larger cohort of SCD patients is recommended and will be crucial in understanding the relationship between COVID-19 and SCD. The pathologic lesions seen at autopsy in our cases are as a result of increased destruction of sickled red cells with the development of anaemia, increased release of haemoglobin and formation of bilirubin, and capillary stasis and thrombosis in SCD patients. Features such as bossing of the frontal and parietal bones of the skull, loose arrangement of the maxillary teeth, long extremities of upper and lower limbs, ulcer at the medial maleolus noticed externally, thinning of cortical bones of the skull, auto-splenectomy, hepatomegaly, pigment stones in the gall bladder, evidence of pulmonary hypertension and cardiomegaly noticed internally, are characteristic and consistent with published findings of SCD at autopsy [13, 16-19]. Also, findings of extra-medullary haematopoiesis, Kupfer cell erythrophagocytosis, thrombi in pulmonary capillaries, fibrosed glomeruli, renal papillary necrosis at histology are known features of SCD [19, 20].

## Conclusion

Postmortems are important investigative tools and should be encouraged to ascertain the real cause of death of SCD patients especially in this pandemic period. Non-diabetics with COVID-19 need to be monitored for elevated blood sugar. Due to the profound effect of SCD on the lungs and the increased risk of VOC, SCD may be an important co-morbidity that needs to be considered in COVID-19 patients and when present should be considered as an adverse risk for poor outcomes. Post discharge thromboprophylaxis and monitoring is recommended. More autopsies are required to fully understand the pathogenesis of COVID-19 in SCD patients so that advances in clinical management can be made. Non-diabetics

### What is known about this topic

- The main pathological effects of COVID-19 infection occur in the lungs as adult respiratory distress syndrome/diffuse alveolar damage with thrombo-embolic phenomena;
- The complications of SCD include vaso-occlusive crises and ACS which primarily affect the lungs;
- SCD patients have characteristic anatomic lesions as a result of sickled red cells with development of anaemia, increased release of haemoglobin and formation of bilirubin, and capillary stasis and thrombosis.

### What this study adds

- SCD patients with COVID-19 (both prothrombotic states) have a high risk for poor outcomes so high level of vigilance is needed while providing care;
- Post-discharge thromboprophylaxis and monitoring of SCD patients with COVID-19 needs to be encouraged;
- Autopsies are capable of reporting evidence of COVID-19 and should be encouraged for presumed cases of SCD deaths.
